# Ecological and health risk assessment of rare earth elements in Kaifeng, Henan Province: a Monte Carlo probabilistic approach

**DOI:** 10.1038/s41598-026-49384-4

**Published:** 2026-05-06

**Authors:** Weichun He, Yuanbo Wang, Keying Chen, Yuling Jiang, Qiang Li, Qin Liang

**Affiliations:** 1 School of Geographic Sciences, Xinyang Normal University, Xinyang, 464000 China; 2 Faculty of Geographical Science and Engineering, Henan University, Kaifeng, 475004 China; 3 School of Design, Shanghai Jiao Tong University, Shanghai, 200240 China; 4 Henan Haoxiang Intellectual Property Service Group Co., Ltd, Zhengzhou, 450000 China

**Keywords:** Kaifeng, Rare earth elements, Monte Carlo model, Risk assessment, Sensitivity analysis, Ecology, Ecology, Environmental sciences, Risk factors

## Abstract

As rare earth elements (REEs) are increasingly used in production and daily life, the threat that REEs in urban soil pose to human health has become increasingly prominent. This study focused on urban soils in Kaifeng City. A total of 70 surface soil samples were collected to determine the concentrations of 13 REEs and conduct a risk assessment. To reduce the impact of uncertainties, Monte Carlo simulation was incorporated into the US EPA model to perform uncertainty analysis for health risk assessment. The results showed that: (1) The average total concentration of REEs in the soil of Kaifeng City was 139.36 mg kg^−1^, and the ecological risk index ranged from 33.98 to 227.01. (2) The average daily doses for adults and children were 0.208 μg kg^−1^ d^−1^ and 1.39 μg kg^−1^ d^−1^, respectively, both of which were far below the reference threshold (70 μg kg^−1^ d^−1^). (3) Monte Carlo simulation revealed that the mean ecological risk index and average daily human exposure dose were similar to the corresponding values obtained from deterministic analysis. The sensitivity contribution rate of exposure parameters was around 80%, remarkably higher than that of REEs concentrations. Consequently, the influence of exposure parameters on the evaluation results should not be underestimated.

## Introduction

Rare earth elements (REEs) consist of 15 lanthanide elements, Sc, and Y, which share similar physicochemical properties. REEs are typically classified into light rare earth elements (LREEs, La to Eu) and heavy rare earth elements (HREEs, Gd to Lu) based on their atomic properties^1^. REEs are widely applied in both high-technology applications and traditional industries. For example, La is used in optical devices, while Nd, due to its unique magnetic properties, is applied in the manufacturing of permanent magnets^2–4^. In recent years, the global demand for REEs has increased exponentially^5^. The global demand for REEs reached 1.51 × 10^5^ tons in 2020, marking a 22.76% increase compared with 2016^6,7^. The wide use and rising production of REEs contribute toward their increasing release into the environment, posing potential risks to biological systems. Thus, REEs have been identified as contaminants of emerging concern^6,8^.

Extensive exploitation and use of rare earth resources have led to increased release of REEs into various environmental compartments, including soil, road dust, atmospheric particles, water, and sediments^9,10^. REEs can enter the human body through multiple exposure pathways, such as ingestion of food and soil, thereby posing potential health risks^11,12^. Several studies have demonstrated that REEs can generally accumulate in the blood, bones, hair^13–15^. When the concentration of REEs in the body beyond a critical threshold can lead to organ dysfunction or metabolic disorders. Wang et al.^16^ found that rural women exposed to high levels of REEs faced an increased risk of hypertension. Similarly, Fan et al.^17^ reported that children chronically exposed to REEs exhibited significantly higher prevalence of intellectual disabilities and impaired physical/neurological development.

Urban soil plays a vital role in the urban ecosystem, significantly influencing urban productivity and residents’ quality of life. To date, current research on health risks associated with urban soil has primarily focused on heavy metals, whereas studies on REEs have been largely limited to REE-contaminated regions. Given the significant variations in urban economic development levels, industrial structures, population densities, and traffic conditions, the potential risks posed by REEs can vary notably across cities. For example, a recent study focusing on the soils of urban parks in Beijing has shown that industrial emissions and traffic activities are the main causes of REEs accumulation^18^. Nevertheless, for cities situated on the Yellow River alluvial plain, investigations specifically targeting REEs accumulation in their soils are still limited. By contrast, recent studies in other regions have shown the value of integrated approaches. These combine contamination indices, source apportionment, and probabilistic risk assessment to evaluate sediment and soil quality in complex environmental settings^19,20^. Thus, Kaifeng was selected as the study area to investigate these risks under specific urban conditions. This study utilized the potential ecological risk index and human health risk assessment approaches to evaluate environmental pollution and health risks associated with REEs. Considering the variability and uncertainty inherent in exposure parameters, Monte Carlo simulation was incorporated to enhance the robustness of the health risk assessment^21^. The findings aim to provide a scientific basis for the management of REEs pollution in urban soils and the safeguarding of human health.

## Materials and methods

### Overview of the study area

Situated at the mid-lower reaches of the Yellow River and the eastern Huanghuai Plain, Kaifeng City ranks among China’s eight ancient capitals. During the 1950s, enterprises focused on chemicals, machinery manufacturing, and non-ferrous metal processing experienced rapid growth. As of early 2025, the city had a population of approximately 4.698 million and covered an area of 6247 km^2^. Its geographical coordinates range from 34° 11′ 45″ N to 35° 01′ 20″ N, and 113° 52′ 15″ E to 115° 15′ 42″ E^22^. Kaifeng exhibits a temperate continental monsoon climate, characterized by an annual average temperature of 14 °C and mean annual precipitation of 628 mm^23^. The soil in Kaifeng exhibits alkaline conditions with an average pH of 8.79 and contains an average organic matter content of 26.29 g/kg^24^. The soil has formed through the combined effects of alluvial deposits from the Yellow River and long-term anthropogenic activities. The soil exhibits a high lime reaction and is primarily composed of loamy and sandy loam. Notably, REEs are prone to adsorption onto soil particle surfaces, which directly influences their environmental behavior and bioavailability.

### Sample collection and pre-treatment

**Fig. 1:**
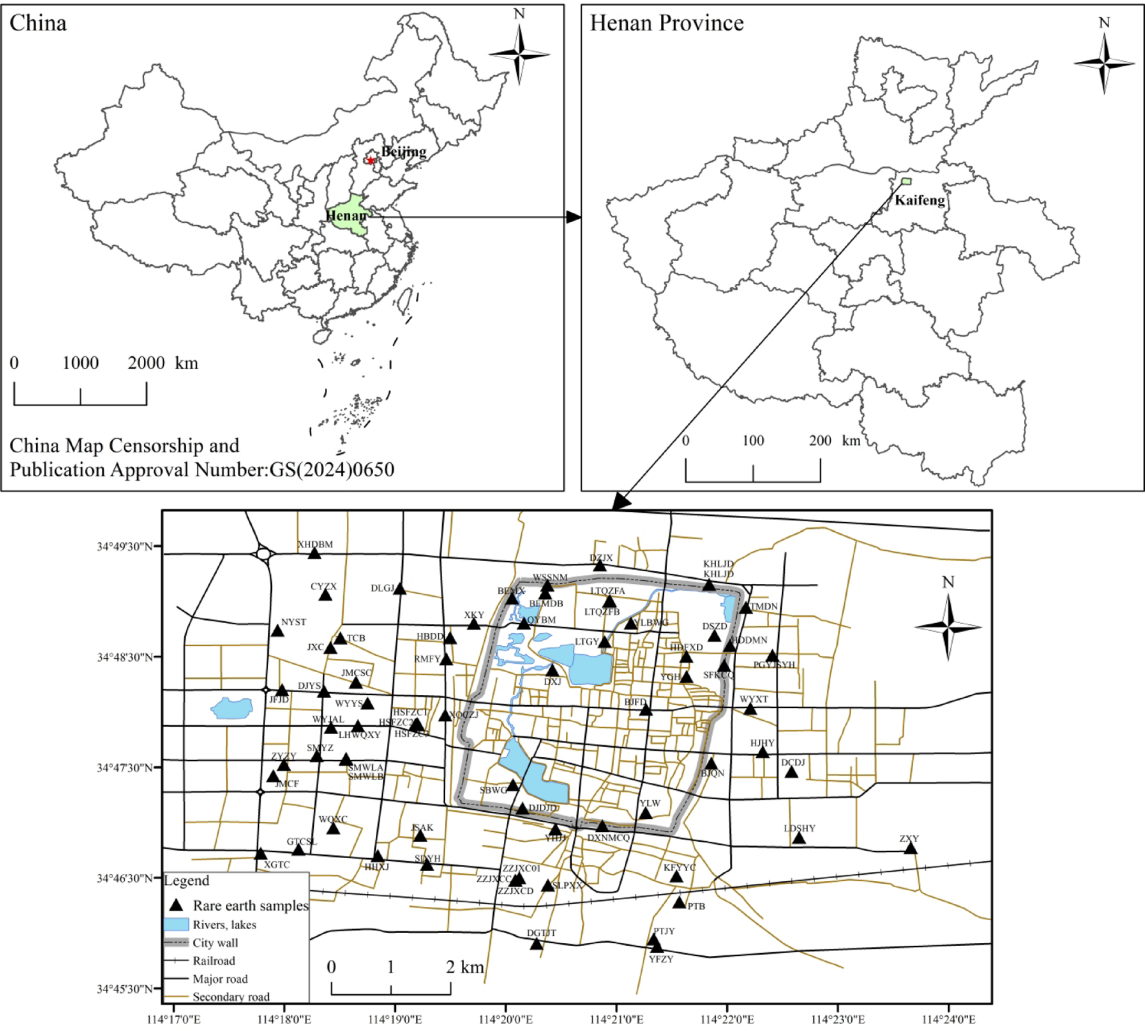
Sampling point distribution in the study area (*n* = 70). National Catalogue Service for Geographic Information of China, National County-level Administrative Boundaries Shapefile Data (https://www.webmap.cn/main.do?method=index). The map was created using ArcGIS 10.8 (Environmental Systems Research Institute, Redlands, CA, USA; https://www.esri.com/).

A total of 70 surface soil samples were collected from urban soils in Kaifeng, as shown in Fig. 1. Surface soil samples were collected using a stainless steel shovel following the quincunx sampling method. All subsamples were thoroughly mixed to form a composite soil sample representative of each sampling site. Soil samples were naturally air-dried at room temperature in the laboratory, followed by removal of stones, organic matter, and other debris. The samples were crushed with a wooden stick and ground in an agate mortar to achieve a particle size passing through a 100-mesh nylon sieve (0.145 mm aperture). The homogenized powders were stored in sealed containers for subsequent analysis. Soil samples were digested with a HNO_3_-HF-HClO_4_ acid system using a fully automatic graphite digestion instrument (HJ/T 166-2004^25^). The concentrations of REEs were then determined by inductively coupled plasma mass spectrometry (ICP-MS, XSeries-2, Thermo Fisher Scientific, USA). For quality control, Rh was used as an internal standard, along with blank samples and soil reference materials (soil sample GSS-2, provided by the National Standard Reference Material Center of China). The recoveries of rare earth elements ranged from 85 to 105%, and the relative standard deviation of parallel samples was below 5%. The method detection limit (LOD) and limit of quantification (LOQ) were determined by analyzing blank samples six times consecutively and calculating three times and ten times the standard deviation, respectively. The LODs and LOQs for La, Ce, Nd, Pr, Sm, Gd, Dy, Eu, Er, Yb, Tb, Tm, and Lu were 0.0007 and 0.002, 0.0016 and 0.0053, 0.0017 and 0.0056, 0.0020 and 0.0067, 0.0010 and 0.0033, 0.0015 and 0.0050, 0.0015 and 0.0050, 0.0015 and 0.0050, 0.0012 and 0.0040, 0.0019 and 0.0063, 0.0010 and 0.0033, 0.0012 and 0.0040, and 0.0010 and 0.0033 µg/kg, respectively.

### Ecological risk assessment

The Hakanson potential ecological risk index was first developed to evaluate the pollution levels of toxic elements in sediments^26^. Its key advantage is that it comprehensively takes into account the toxic effects of pollutants and environmental background values. This allows for the quantification of the potential harm pollutants pose to ecosystems. Originally designed for aquatic sediments, this index has been increasingly used in recent years to assess the ecological risks of pollutants like rare earth elements and heavy metals in soil^27–30^. Multiple relevant studies have confirmed its applicability and rationality in soil ecological risk assessment. Ecological risk assessment primarily involves calculating element-specific toxicity response coefficients. However, limited availability of literature data on calculating toxicity response coefficients for REEs poses significant challenges to conducting comprehensive ecological risk assessments. In this study, toxicity response coefficients for REEs were adopted from Chen et al.^31^, with values as follows: La = 1, Ce = 1, Pr = 5, Nd = 2, Sm = 5, Eu = 10, Gd = 5, Tb = 10, Dy = 5, Er = 5, Tm = 10, Yb = 5, Lu = 20. These coefficients have been used in recent REE studies^32,33^. The ecological risk of REEs is calculated as follows:

1 $$\begin{array}{*{20}c} {E_{r}^{i} = T_{r}^{i} \times \frac{{C_{a}^{i} }}{{C_{{a_{0} }}^{i} }}} \\ \end{array}$$

2 $$\begin{array}{*{20}c} {RI = \mathop \sum \limits_{i = 1}^{n} E_{r}^{i} } \\ \end{array}$$

where *RI* is the comprehensive ecological risk index of REEs, ${E}_{r}^{i}$ is the ecological risk factor for element *i*, ${T}_{r}^{i}$ is the toxicity response coefficient of element *i*, ${C}_{a}^{i}$ is the measured concentration of element *i* in the soils, ${C}_{{a}_{0}}^{i}$ is the background concentration of element *i*. The rare earth element background values are derived from our team’s prior study^24^, based on the concentrations measured at control sampling sites in Kaifeng City.

### Health risk assessment

Health risk assessment is a systematic framework for evaluating environmental pollutants and quantifying their potential harm to human health and ecosystems^34^. Using the health risk assessment framework established by the United States Environmental Protection Agency (US EPA), this study conducted a quantitative evaluation of REEs exposure doses for Kaifeng’s urban population via multiple exposure pathways. Physiological and behavioral differences between children and adults lead to varying exposure risks under identical environmental conditions. The specific formula is as follows.

3 $$\begin{array}{*{20}c} {ADD_{ing} = \frac{{C_{i} \times IngR \times ED \times EF \times CF}}{BW \times AT}} \\ \end{array}$$

where *C*_*i*_ is the concentration of rare earth element *i* in soil (mg kg^−1^), *ADD*_*ing*_ is the average daily dose of element *i* (μg·kg^−1^ d^−1^), the other parameters are fully presented in Table 1. Based on previous studies^18,35–39^, a daily permissible intake of 70 μg kg^−1^ d^−1^ for REEs is considered safe for humans, and this reference threshold was adopted for health risk assessment in this study.

**Table 1:** Exposure parameters for health risk evaluation based on Monte Carlo simulation.

Parameters symbols	Exposure parameters	Reference values		Probabilistic distribution	Source
		Adults	Children		
*IngR*	Soil ingestion rate/mg d^−1^	100	200	TR (70,100,200)	^40,41^
*ED*	exposure duration/a	24	6		^41^
*EF*	Annual exposure frequency/d·a^−1^	350	350	UN (120,365)	^40,42^
*CF*	Unit conversion factor/kg mg^−1^	1 × 10^–6^	1 × 10^–6^		^41^
*BW*	Body weight/kg	61.8	19.2	LN (59.78,1.07) (adults) LN (16.68,1.48) (children)	^43,44^
*AT*	Average time of exposure to contaminated soils/d	9125	2190		^27^

TR, Triangular distribution (minimum, most probable, maximum); UN, Uniform distribution (minimum, maximum); LN, Lognormal Distribution (mean, standard deviation).

### Data processing and analysis

Statistical analysis was performed using Excel 2021 and SPSS 26, while uncertainty analysis for potential ecological risks, human health risks, and exposure parameters was conducted with Oracle Crystal Ball 11.1.30. Data processing and visualization were completed using Origin 2022.

## Results and discussion

### Descriptive statistics for REEs in Kaifeng soil

**Fig. 2:**
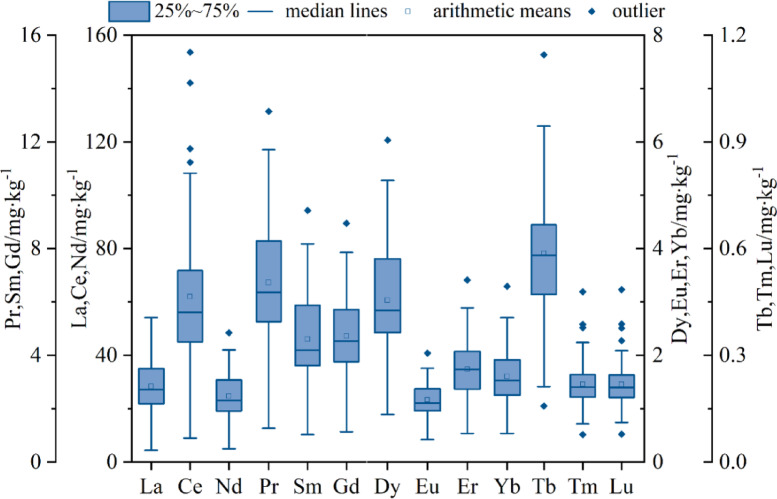
Box plot of REEs concentrations in urban soils of Kaifeng (*n* = 70).

The average concentrations of REEs in Kaifeng soil (mg kg^−1^) were ranked as: Ce (61.88) > La (28.31) > Nd (24.57) > Pr (6.73) > Gd (4.71) > Sm (4.61) > Dy (3.03) > Er (1.74) > Yb (1.60) > Eu (1.16) > Tb (0.59) > Lu (0.22) = Tm (0.22) (Fig. 2). This sequence significantly differed from the upper continental crust (UCC^45^) (Ce > La > Nd > Pr > Sm > Gd > Dy > Er > Yb > Eu > Yb > Eu > Tb > Tm > Lu), indicating that elemental distributions in the study area were influenced by external sources. In a box plot, the box represents the interquartile range (IQR), and the horizontal line indicates the median. The whiskers extend to 1.5 times the IQR, and values beyond the whiskers are considered outliers^46^. Among all 13 rare earth elements, only lanthanum (La) concentrations exhibited no outliers, as illustrated in Fig. 2. Most box plots exhibited asymmetric whisker lengths, indicating skewed distributions. Medians of most REE concentrations did not align with mean values, suggesting non-normal data distributions.

Total concentrations of 13 REEs in the surface soil of Kaifeng ranged from 24.44 to 304.48 mg kg^−1^, averaging 139.36 mg kg^−1^ (Table 2). The average total REEs concentration in Kaifeng’s surface soil was significantly lower than the background concentrations of Beijing soil, the Chinese national soil background (CBV), upper continental crust (UCC), and North American Shale Composite (NASC). While most REEs showed concentrations below UCC levels, Sm, Eu, and Gd exceeded their respective UCC background values by 2.44%, 31.32%, and 23.95%. Maximum concentrations of individual REEs exceeded China’s national soil averages, indicating significant enrichment of REEs in the study area.

**Table 2:** Statistical description of REEs in surface soil in Kaifeng city (mg kg^−1^).

REEs	Mean	Med	Min	Max	SD	CV (%)	Kaifeng control area^24^	Beijing^48^	CBV^49^	UCC^45^	NASC^50,51^
La	28.31	27.10	4.43	54.15	9.26	32.71	22.88	30.59	39.7	30	31.1
Ce	61.88	56.09	8.90	153.56	26.26	42.44	47.82	59.71	68.4	64	66.7
Pr	6.73	6.35	1.27	13.14	2.20	32.63	5.38	6.82	7.17	7.1	7.90
Nd	24.57	23.09	5.00	48.38	8.01	32.58	19.29	25.89	26.40	26	27.4
Sm	4.61	4.19	1.03	9.43	1.55	33.58	3.47	4.76	5.22	4.5	5.59
Eu	1.16	1.10	0.42	2.04	0.29	25.04	0.97	1.08	1.03	0.88	1.18
Gd	4.71	4.54	1.13	8.95	1.37	28.97	3.68	4.59	4.60	3.8	5.20
Tb	0.59	0.58	0.16	1.14	0.16	27.79	0.47	0.62	0.63	0.64	0.85
Dy	3.03	2.84	0.89	6.03	0.96	31.77	2.17	3.31	4.13	3.5	5.75
Er	1.74	1.73	0.53	3.41	0.52	30.09	1.24	1.92	2.54	2.3	3.40
Tm	0.22	0.21	0.08	0.48	0.06	29.39	0.16	0.29	0.37	0.33	0.50
Yb	1.60	1.53	0.53	3.29	0.51	31.77	1.10	1.8	2.44	2.2	3.06
Lu	0.22	0.21	0.08	0.48	0.06	29.84	0.16	0.27	0.36	0.32	0.46
$\sum REE$	139.36	129.12	24.44	304.48	49.87	35.78	108.79	141.65	162.99	145.57	159.09

LREEs concentrations in soil samples ranged from 21.04 to 280.69 mg kg^−1^, accounting for approximately 91.31% of the $\sum REEs$, indicating pronounced enrichment of LREEs. The coefficient of variation (CV) is a statistical metric used to quantify data dispersion and indicate heterogeneity within the study area^47^. The coefficients of variation for the REEs in Kaifeng followed the order: Ce (42.44%) > Sm (33.58%) > La (32.71%) > Pr (32.63%) > Nd (32.58%) > Yb (31.77%) = Dy (31.77%) > Er (30.09%) > Lu (29.84%) > Tm (29.39%) > Gd (28.97%) > Tb (27.79%) > Eu (25.04%) (Table 2). Ce showed the highest spatial heterogeneity (42.44%), likely influenced by anthropogenic activities^8^, while Eu exhibited minimal variability and stable spatial distribution.

### Potential ecological risk assessment of REEs

The background values of rare earth elements in Kaifeng soil were adopted as the reference threshold to calculate ecological risk indices for 13 REEs (La, Ce, Pr, Nd, Sm, Eu, Gd, Tb, Dy, Er, Tm, Yb, Lu) at each sampling site in Kaifeng^24^. Due to differences in the number of REEs analyzed compared to Chen et al.^31^, both the combined risk index (*RI*) and potential ecological risk factor (${E}_{r}^{i}$) grading criteria were recalibrated. As established by related studies^52–54^, ${E}_{r}^{i}$ is defined as the maximum toxicity coefficient among the contaminants, with upper threshold values for other risk levels derived by multiplying the preceding level’s upper limit by 2. In this study, the maximum toxicity coefficient of the 13 REEs is 20, so the upper limit value for “low risk” is defined as 20, and the remaining values are multiplied by 2 to obtain the grading criteria of ${E}_{r}^{i}$. The adjustment of the *RI* is as follows: $150\times (84/133)\approx 95$. Other classification values are multiplied by 2, as shown in Table 3. Furthermore, the ecological risk assessment method used in this study incorporated more elements compared to Hakanson’s research^26^ to avoid underestimating or overestimating the ecological risks of REEs.

**Table 3:** Classification of ecological risk corresponding to ${E}_{r}^{i}$ and *RI.*

${E}_{r}^{i}$		Risk degree	*RI*		Risk degree
Hakanson	This study		Hakanson	This study	
< 40	< 20	Low	< 150	< 95	Low
40 ~ 80	20 ~ 40	Moderate	150 ~ 300	95 ~ 190	Moderate
80 ~ 160	40 ~ 80	Strength	300 ~ 600	190 ~ 380	Strength
160 ~ 320	80 ~ 160	Strong	≥ 600	≥ 380	Strong
≥ 320	≥ 160	Very strong			

**Fig. 3:**
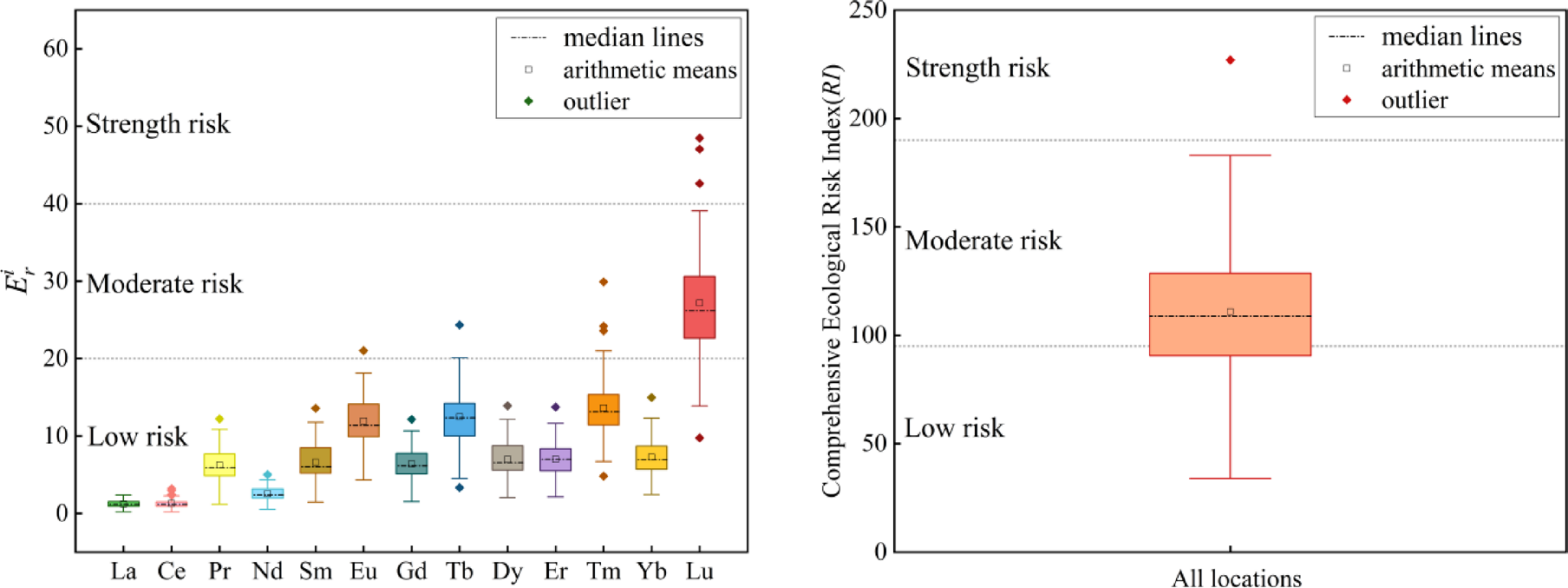
${E}_{r}^{i}$ and *RI* values of REEs in urban soils of Kaifeng city (*n* = 70).

The potential ecological risk values for REEs were as follows: La (0.19–2.37) < Ce (0.19–3.21) < Nd (0.52–5.02) < Pr (1.18–12.21) < Gd (1.53–12.16) < Sm (1.48–13.58) < Dy (2.06–13.89) < Er (2.15–13.74) < Yb (2.42–14.96) < Eu (4.33–21.03) < Tb (3.34–24.35) < Tm (4.83–29.91) < Lu (9.77–60.57) (Table 4). As shown in Fig. 3 and Table 4, at most sampling sites, the ${E}_{r}^{i}$ values of most REEs were below 20, indicating low ecological risk. However, the maximum ${E}_{r}^{i}$ values of Eu (21.03), Tb (24.35), Tm (29.91), and Lu (60.57) exceeded the low-risk threshold (20). Specifically, Eu, Tb, and Tm fell into the moderate-risk level (20 ~ 40), while Lu reached the strong-risk level (40 ~ 80), suggesting localized ecological risks from specific REEs at certain sites. The comprehensive ecological risk index (*RI*) of REEs averaged 110.93 (range: 33.98–227.01). The mean *RI* indicates a moderate risk level (95 ~ 190), and the maximum *RI* falls into the strong-risk level (190 ~ 380), reflecting an overall moderate ecological risk in Kaifeng urban soils, with strong risk at individual sites requiring further attention. Among the analyzed REEs, Lu contributed most significantly to the *RI* at 24.51%, followed by Tm (12.30%) and Tb (11.26%). Conversely, La displayed the lowest contribution (1.12%).

**Table 4:** ${E}_{r}^{i}$ and *RI* values of REEs in soils (*n* = 70).

	La	Ce	Pr	Nd	Sm	Eu	Gd	Tb	Dy	Er	Tm	Yb	Lu	*RI*
Mean	1.24	1.29	6.25	2.55	6.64	11.96	6.40	12.49	6.98	7.00	13.65	7.28	27.19	110.93
Median	1.18	1.17	5.90	2.39	6.03	11.37	6.16	12.36	6.55	6.98	13.14	6.95	26.20	108.81
Min	0.19	0.19	1.18	0.52	1.48	4.33	1.53	3.34	2.06	2.15	4.83	2.42	9.77	33.98
Max	2.37	3.21	12.21	5.02	13.58	21.03	12.16	24.35	13.89	13.74	29.91	14.96	60.57	227.01

Lu made the highest contribution, which may be attributed to its largest toxicity coefficient. A comparison between sampling sites and risk levels revealed that the higher comprehensive risk at certain sites may be closely related to the regional environmental conditions. In the urban soils of Kaifeng City, most sampling sites with elevated rare earth element risks were located in the southeastern corner. As a traditional industrial and shipping center, this area hosts facilities such as thermal power plants, fertilizer plants, and boiler plants. Industrial dust, particularly fly ash from thermal power plants and vehicle exhaust, serves as the primary source of rare earth elements in this region^24^.

### Human health risk assessment

This study applied the US EPA health risk assessment model, incorporating REEs concentrations and soil exposure parameters, to calculate the average daily dose (ADD) of REEs for urban residents exposed to Kaifeng soil. The results indicated that the ADD for adults and children were 0.208 μg kg^−1^ d^−1^ and 1.39 μg kg^−1^ d^−1^, respectively. Children exhibited slightly higher dose levels than adults, which may be attributed to their more frequent hand-to-mouth and hand-to-object contact behaviors. Children demonstrated higher sensitivity to pollutants via the ingestion pathway and posed greater health risks compared to adults. This finding aligns with previous health risk assessment results for different age groups reported by Lian et al.^36^ and Liu et al.^18^. With the human daily intake threshold for REEs and the subclinical damage threshold established at 70 μg·kg^−1^ d^−1^ and 100–110 μg·kg^−1^ d^−1^^13^, respectively, both of these limits are substantially higher than the calculated ADD in the study area, indicating that the current REEs exposure levels pose a low health risk. However, REEs can enter the human body through multiple exposure routes, including dermal absorption, inhalation, and hand-to-mouth contact, posing potential risks to endocrine, nervous, and other physiological systems^15^. Therefore, the continuous monitoring of REEs daily exposure levels is essential to assess associated health risks.

**Fig. 4:**
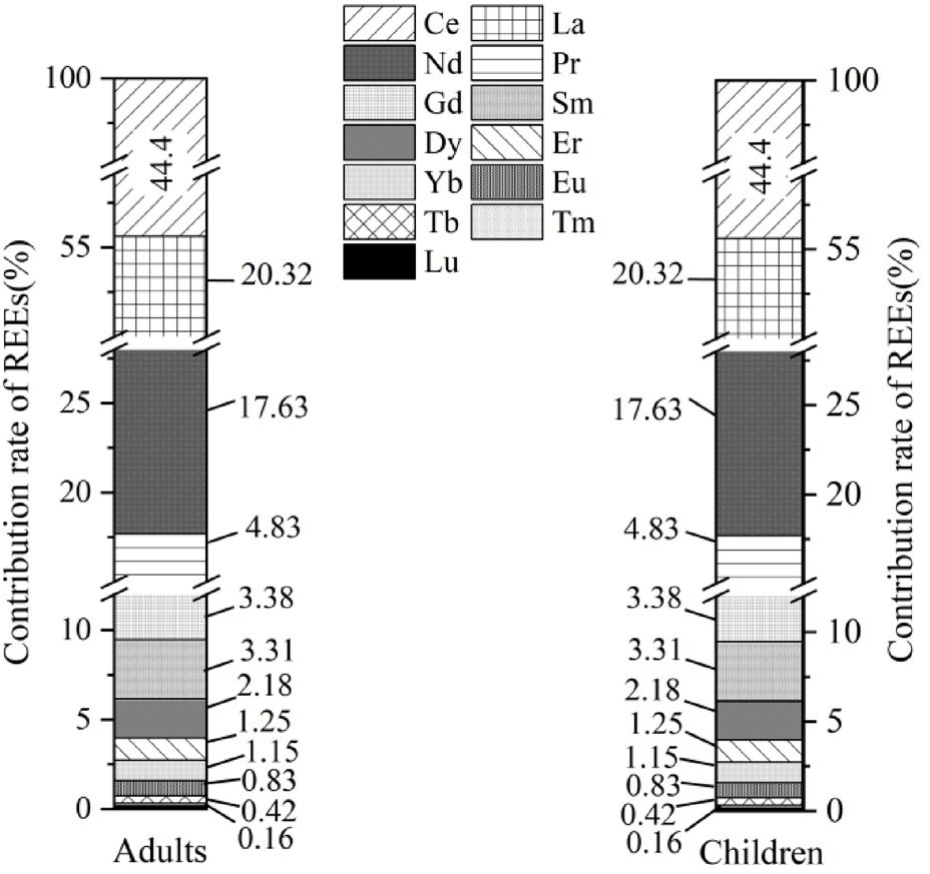
Evaluation results of the main contributing elements (*n* = 70).

LREEs accounted for 91.31% of human health risks, significantly higher than HREEs (8.69%). Although children exhibited slightly higher daily exposure doses compared to adults, risk contribution rates remained consistent across age groups. Element contribution rates (Fig. 4) revealed that Ce contributed most significantly to human health risks at 44.40%, followed by La (20.32%) and Nd (17.63%). Conversely, Tm and Lu exhibited the lowest contributions, both at 0.16%. These findings identified Ce, La, and Nd as the primary contributors to human health risks. It is important to note that this study solely quantified the exposure doses through soil ingestion and did not account for dermal contact or inhalation pathways. This approach might lead to an underestimation of the overall exposure^55–57^, especially in the case of children, which is a potential limitation of the present study.

### Uncertainty assessment

#### Uncertainty assessment of potential ecological risk

**Fig. 5:**
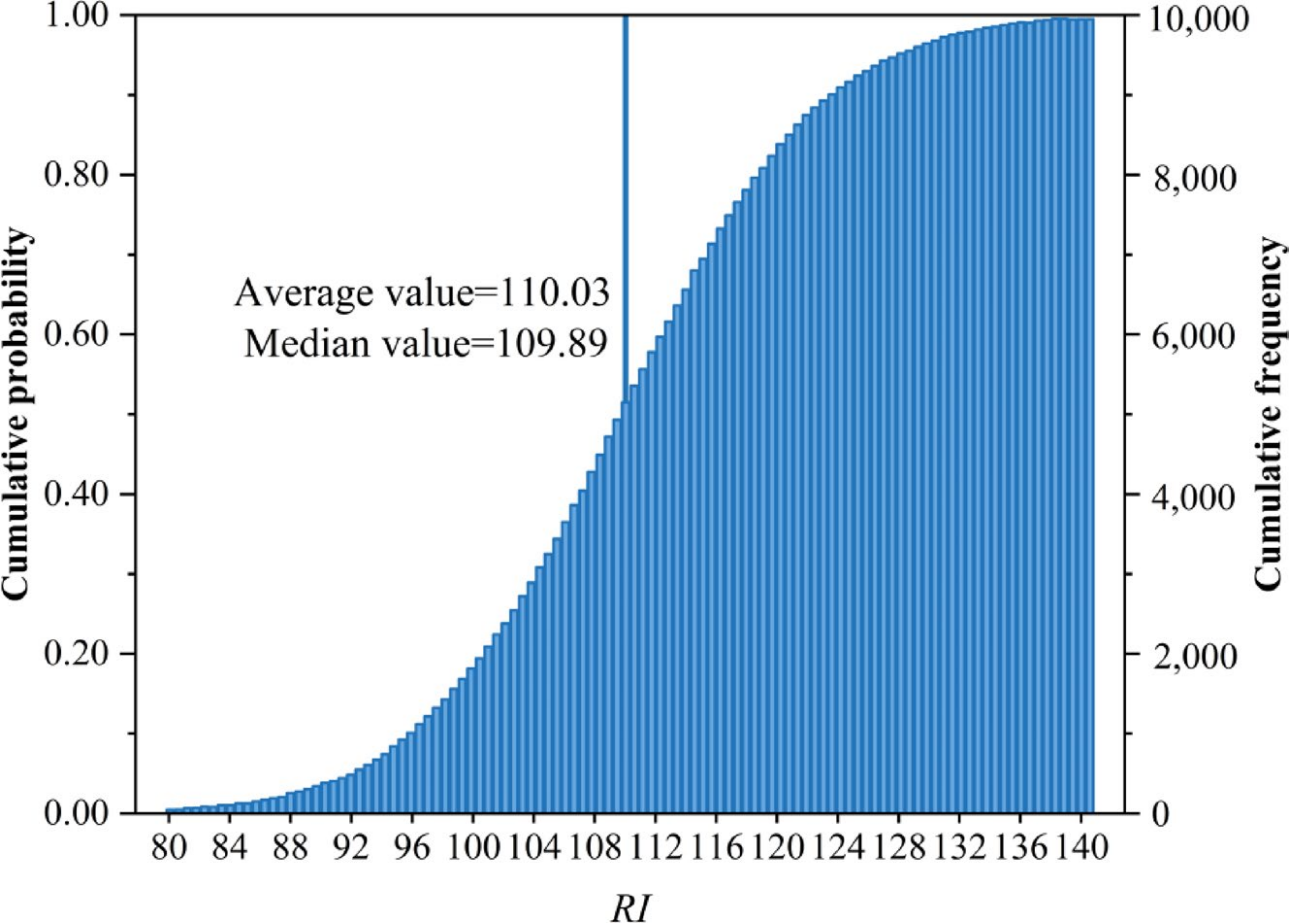
Cumulative frequency and probability distribution of ecological risk of REEs (*n* = 70).

Probabilistic distribution of ecological risk indices provides a comprehensive understanding of REE ecological risks in Kaifeng’s surface soil, given the limitations of soil sampling in capturing risks across broader spatial scales^58^. Anderson–Darling tests were applied to determine the distribution model of potential ecological risk indices, followed by 10,000 iterations of risk index simulations using Oracle Crystal Ball 11.1.30 to generate the cumulative probability diagram (Fig. 5). Simulation results yielded a median of 109.89 and a mean of 110.03, which were in close agreement with deterministic ecological risk index values (108.81 and 110.93, respectively), demonstrating consistency between the two approaches. The 10%, 50%, 90%, and 100% evaluated *RI* values for REEs were 96.16, 109.89, 123.82, and 159.01, respectively, all of which exceeded the low-risk threshold of 95 and remained within the moderate-risk level (95 ~ 190). This confirmed predominantly moderate ecological risks in the study area. However, due to the limited representativeness of sampling data, REEs merit continued attention to address potential uncertainties in risk extrapolation.

#### Uncertainty assessment of human health risk

Health risk assessment outcomes for human exposure to REEs depend on sample representativeness, sampling randomness, and exposure parameter accuracy^59^. Anderson–Darling goodness-of-fit tests indicated that La, Gd, and Er follow Gamma distributions; Ce fits a Generalized Extreme Value distribution; Dy follows a Beta distribution; Tb, Tm, and Lu align with Logistic distributions; and remaining REEs follow Lognormal distributions. Fitted distributions of exposure parameters were derived from the relevant literature^40,44^: IngR follows a Triangular distribution (70, 100, 200); EF follows a Uniform distribution (120, 365); children’s body weight (BW) follows a Lognormal distribution (16.68, 1.48); and adults’ BW follows a Lognormal distribution (59.78, 1.07) (Table 1).

**Fig. 6:**
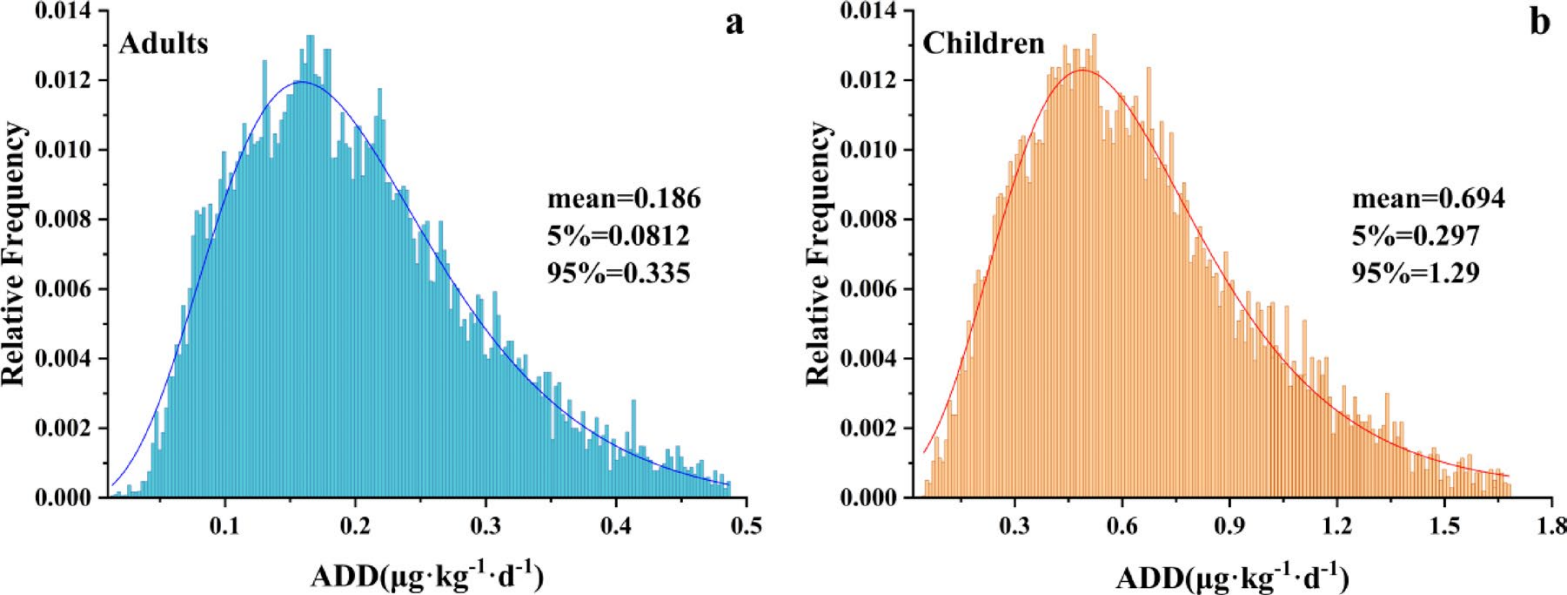
Average daily dose and probability distribution of REEs for adults (a) and children (b) (*n* = 70).

Monte Carlo simulations with 10,000 iterations were performed using Crystal Ball to estimate daily REEs exposure doses in surface soil of Kaifeng, based on probability distributions of sampling data and exposure parameters. Results included average daily doses for adults and children, along with their probability distribution diagrams (Fig. 6). Results showed mean ADD values of 0.186 μg kg^−1^ d^−1^for adults and 0.694 μg kg^−1^ d^−1^ for children, with 95% evaluated ADD values of 0.334 μg kg^−1^ d^−1^ and 1.28 μg kg^−1^ d^−1^, respectively. Notably, all values were significantly lower than the health safety threshold of 70 μg kg^−1^ d^−1^. The Monte Carlo simulation enhanced the robustness of the health risk assessment by accounting for the variability and uncertainty in exposure parameters, further supporting the conclusion that there is no significant health risk under the current exposure scenario^21^.

#### Sensitivity analysis

**Fig. 7:**
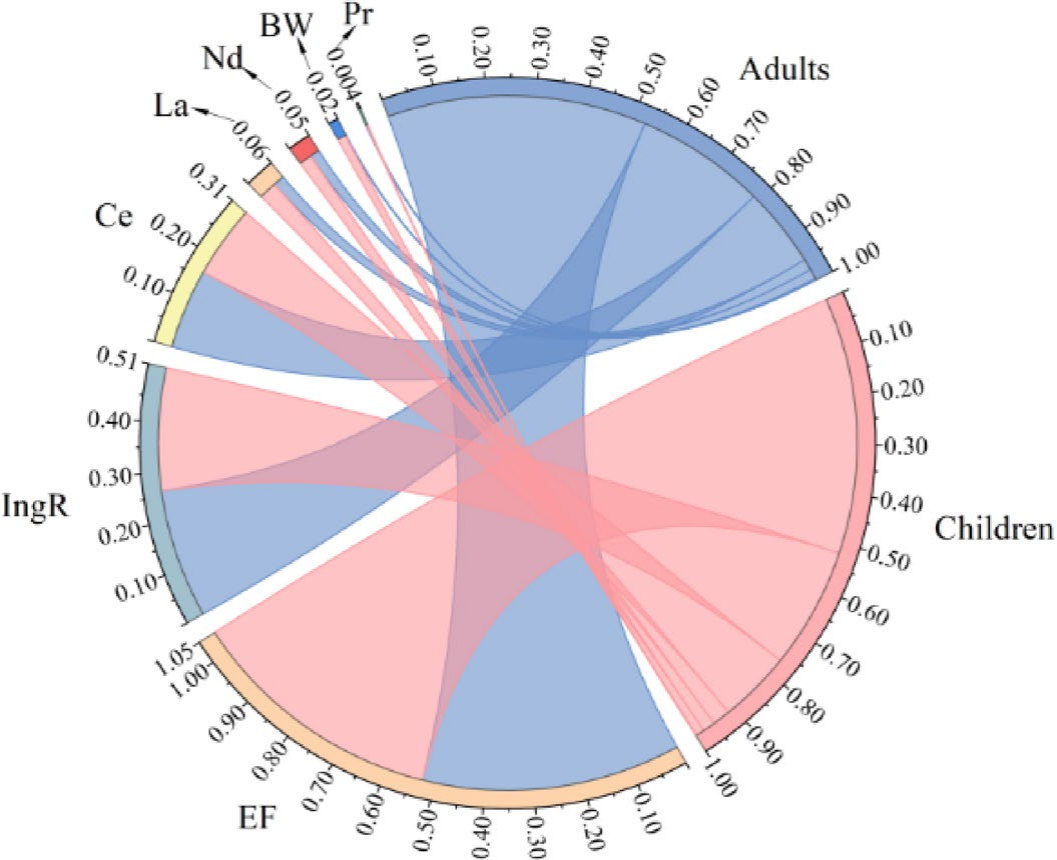
Sensitivity analysis of health risk for adults and children (*n* = 70).

Sensitivity analysis was conducted to determine the influence of rare earth element concentrations and exposure parameters on risk assessment outcomes. Higher contribution rates indicated stronger influences^60,61^. Sensitivity analysis results generated by Crystal Ball (Fig. 7) indicated that REEs accounted for approximately 20.97% and 19.57% of total sensitivity for adults and children, respectively. Among REEs, Ce showed the highest sensitivity contributions (16.32% for adults, 14.73% for children), followed by La (2.31% for adults, 2.53% for children) and Nd. Exposure frequency (*EF*) was the key exposure parameter affecting health risks for adults (52.55%) and children (52.14%), while soil ingestion rate (*IngR*) sensitivity accounted for 26.27% and 24.66%, respectively. Body weight (*BW*) exhibited the lowest sensitivity. Total contribution of exposure parameters in sensitivity analysis accounted for approximately 80%, significantly higher than threefold that of REE concentrations. Previous research^61^ highlighted that when total exposure parameter sensitivity exceeds three times element concentration sensitivity, their influence on health risk assessment should not be overlooked. Thus, *EF*, *IngR*, and Ce concentrations in Kaifeng soils emerged as critical parameters governing health risks via soil ingestion pathways. It should be noted that exposure parameters such as *EF* and *IngR* are mathematically linearly related to ADD in the model structure, which partly contributes to their high sensitivity. Nonetheless, this finding is consistent with recent studies that also identified exposure parameters as dominant contributors to health risk variability in similar environmental contexts^18,62^. Monte Carlo simulations improve the reliability of health risk assessment results, while sensitivity analysis provides insights into parameter contributions and facilitates targeted mitigation strategies for REEs hazards.

## Conclusions

1. Total REEs in Kaifeng’s surface soils averaged 139.36 mg kg^−1^. Among 13 analyzed REEs, only Sm, Eu, and Gd exceeded UCC values by 2.44%, 31.32%, and 23.95%, respectively. Notably, Ce exhibited the highest coefficient of variation, reflecting significant spatial variability potentially linked to anthropogenic inputs.
2. The *RI* values for REEs in Kaifeng soils ranged from 33.98 to 227.01, indicating moderate risks in certain samples. Mean daily REEs intakes for adults and children were 0.208 μg kg^−1^ d^−1^ and 1.39 μg kg^−1^ d^−1^, respectively, both below the safety limit of 70 μg kg^−1^ d^−1^. Children demonstrated higher health risks from REEs compared to adults, a finding meriting attention.
3. Monte Carlo simulations revealed minor discrepancies between mean potential ecological risk values and deterministic analysis results for both adults and children. Sensitivity analysis identified *EF*, *IngR*, and Ce concentrations as critical determinants of health risks, underscoring their importance in risk assessment frameworks.

## Data Availability

Data will be made available on request.
